# Resting state neurophysiology of agonist-antagonist myoneural interface in persons with transtibial amputation

**DOI:** 10.21203/rs.3.rs-2362961/v1

**Published:** 2023-02-09

**Authors:** Laura Chicos, D. Rangaprakash, Robert Barry, Hugh Herr

**Affiliations:** MIT; Massachusetts General Hospital Harvard Medical School; Massachusetts General Hospital & Harvard Medical School; Massachusetts Institute of Technology

**Keywords:** Transtibial amputation, fMRI, Brain network reorganization, Functional connectivity, Neuroimaging, Limb loss

## Abstract

The agonist-antagonist myoneural interface (AMI) is a novel amputation surgery that preserves sensorimotor signaling mechanisms of the central-peripheral nervous systems. Our first neuroimaging study investigating AMI subjects (*Srinivasan et al., Sci. Transl. Med. 2020*) focused on task-based neural signatures, and showed evidence of proprioceptive feedback to the central nervous system. The study of resting state neural activity helps non-invasively characterize the neural patterns that prime task response. In this first study on resting state fMRI in AMI subjects, we compared resting state functional connectivity in patients with transtibial AMI (n=12) and traditional (n=7) amputations, as well as biologically intact control subjects (n=10). We hypothesized that the AMI surgery will induce functional network reorganization that significantly differs from the traditional amputation surgery and also more closely resembles the neural configuration of controls. We found AMI subjects to have lower connectivity with salience and motor seed regions compared to traditional amputees. Additionally, with connections affected in traditional amputees, AMI subjects exhibited a connectivity pattern more closely resembling controls. Lastly, sensorimotor connectivity in amputee cohorts was significantly associated with phantom sensation (R^2^=0.7, *p*=0.0008). These findings provide researchers and clinicians with a critical mechanistic understanding of the effects of the AMI surgery on the brain at rest, spearheading future research towards improved prosthetic control and embodiment.

## One-sentence Summary

Transtibial amputee subjects with the agonist-antagonist myoneural interface surgery show spontaneous brain network patterns more closely resembling biologically-intact controls compared to subjects with traditional amputation.

## Introduction

Every year in the United States, approximately 150,000 patients undergo lower-extremity amputations due to reasons such as diabetes mellitus, peripheral vascular disease, neuropathy, and trauma (1). The financial cost of lower-extremity amputation in the U.S. due to dysvascular etiology alone, which accounts for the majority (82%) of amputations, exceeds $4.3 billion annually (2, 3). Over 50% of all amputations are at the transtibial level, or below the knee (4). The traditional surgical technique employed for nearly all of these amputations involves transecting nerves and burying them within the residuum. This traditional amputation (TA) often leaves patients with painful neuromas, phantom limb pain, and pathological brain activity (5, 6). One critical drawback with the TA is that agonist-antagonist muscle pairs are cleaved, which limits muscle spindle and Golgi tendon organ-based afferent proprioceptive signals from reaching the central nervous system (CNS). Subsequently, patients rely mostly on visual feedback to guide their prosthesis during free-space movements instead of natural sensory afferent feedback. While there is still no clear mechanistic understanding as to how sensorimotor phantom limb representation modulates pain processing, the leading hypothesis for decades has been that phantom limb pain may be the result of maladaptive plasticity in response to the loss of afferent input.

**Figure 1:**
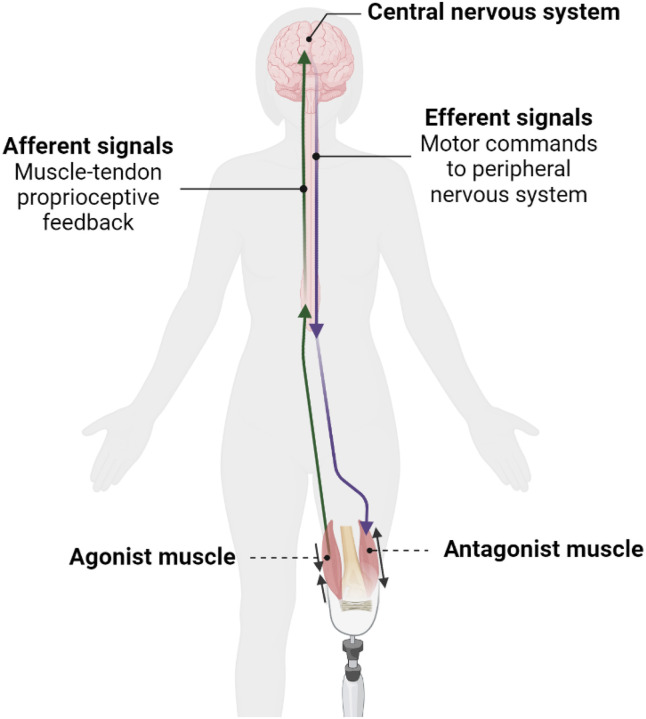
The Agonist-Antagonist Myoneural Interface (AMI) amputation architecture. An agonist-antagonist muscle pair is surgically created in the residual limb for coupled fascicle dynamics. The purple arrow represents the pathway of efferent signals that are involved in transmitting motor commands instantiated in the central nervous system to the peripheral nervous system to initiate and guide movement. The green arrow represents the pathway of afferent signals involved in communicating muscle-tendon proprioceptive feedback from the residual limb to the central nervous system.

The agonist-antagonist myoneural interface (AMI) amputation promotes physiological function of the central-peripheral sensorimotor signaling mechanisms by reestablishing agonist-antagonist muscle pairs (Fig. 1) (7). The effect of the restoration of afferent feedback due to the AMI amputation on CNS plasticity in relation to prosthesis embodiment, as well as phantom limb sensation and pain, are potentially impactful research areas.

The first neuroimaging study investigating AMI subjects focused on task-based neural signatures, and showed evidence of AMI promoting proprioceptive feedback to the CNS in terms of functional activation in proprioceptive centers compared to persons with TA (8). Subjects with AMI amputation had higher position differentiation task scores than TA subjects on tasks requiring motor control and proprioception, presumably due to the reinstituted afferent feedback. Furthermore, the strongly coupled relationship between the sensorimotor network and the visual cortex was associated with subjects that reported weaker non-painful phantom sensations. Building on these important discoveries, it is also important to gain a mechanistic understanding of how AMI affects intrinsic brain networks and plasticity at rest, to further substantiate the efficacy of the AMI surgical architecture and its overall impact on the brain, prosthesis control, embodiment and quality of life.

Resting state fMRI (rsfMRI), or imaging during a task-free state, is an important tool for understanding neurophysiological states without confounding variability from task compliance or performance within and between subjects. RsfMRI helps in non-invasively probing intrinsic, spontaneous functional networks by quantifying co-fluctuations in the blood oxygenation level-dependent (BOLD) signal between two spatially-distinct regions of interest (ROI), which is termed as functional connectivity (FC) (9). These correlated fluctuations are most prominent at low frequencies (< 0.1 Hz) and prime task response (10). There have been a few rsfMRI studies examining brain network-level functional reorganization after TA; for instance, one study showed changes in FC between sensorimotor and subcortical structures over time since amputation (11, 12). However, there are no existing studies on resting state FC signatures in transtibial AMI and their differences with TA or biologically-intact control subjects. We address this critical gap in this study. We hypothesized that the AMI amputation induces neuroplastic reorganization of functional networks that both significantly varies from the TA, and that also bears closer resemblance to biologically-intact controls in terms of FC maps. To test these hypotheses, we employed a combination of standard as well as robust novel fMRI processing methods to investigate seed-based connectivity (SBC) in AMI, and compare with SBC in TA and control groups.

## Results

**Figure 2:**
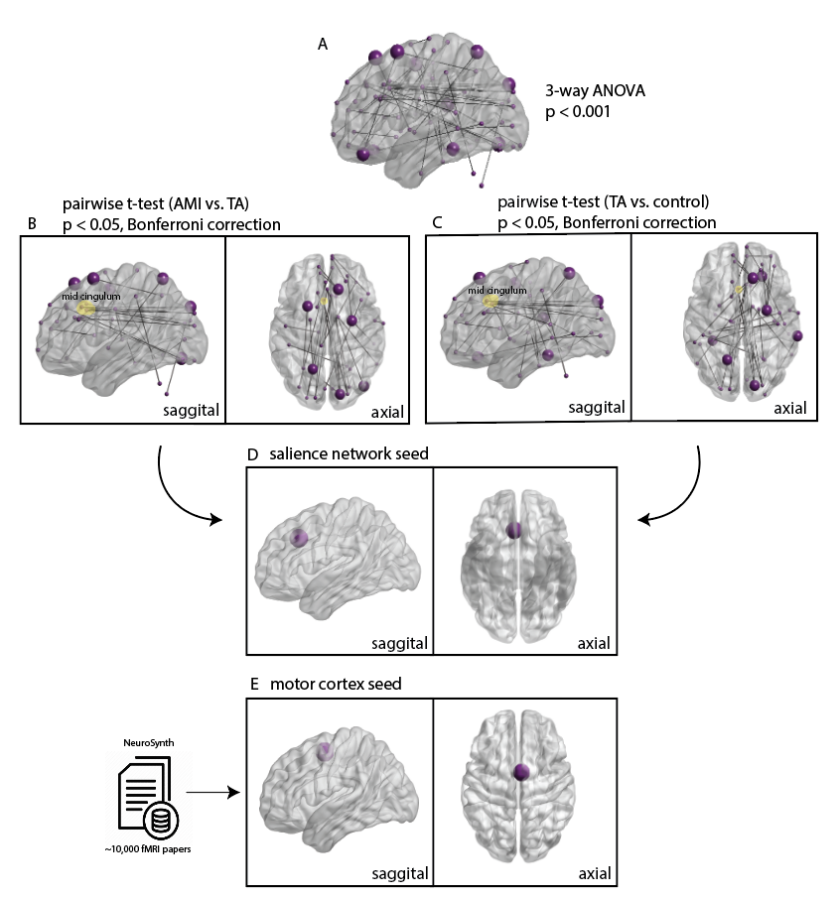
Whole-Brain Exploratory Analysis and ROIs selected for further investigation in a seed-based connectivity analysis. **(A)** Significant connections after a three-way ANOVA (p^unc^ < 0.001) between all three subject conditions. **(B)** Significant pairwise connections (p < 0.05, Bonferroni correction) between AMI and TA groups from the subset of significant connections after the three way ANOVA. **(C)** Significant pairwise connections (p < 0.05, Bonferroni correction) between TA and control groups from the subset of significant connections after the three way ANOVA. The node highlighted in the mid-cingulum represents the seed region in the salience network. **(D)** The salience network ROI (spherical radius = 10mm) chosen due to its uniquely high node degree and connectivity in both AMI vs. TA and TA vs. control pairwise connections. (E) The primary motor cortex ROI (spherical radius = 10mm) as defined by a meta-analysis of neuroimaging studies in Neurosynth.

Our goal was to investigate SBC in AMI, but, since there have been no prior rsfMRI studies on AMI to help us identify seed regions, we assayed the rsfMRI data in an exploratory whole-brain analysis first. To highlight the timeseries that were significantly correlated among any pair of the three subject conditions, a three-way ANOVA (p^unc^ < 0.001) was performed (Fig. 2A). Pairwise t-tests (p < 0.05, Bonferroni correction) were then performed between each subject condition to determine a seed region according to the following criteria: node degree and implication in more than one pairwise comparison. A region located on the mid cingulum and part of the salience network (Fig. 2D) was uniquely identified as having the highest node degree and connectivity in pairwise comparisons between AMI and TA subjects (Fig. 2B), as well as TA and control subjects (Fig. 2C). A region in the medial primary motor cortex (Fig. 2E) was manually chosen for further investigation due to its clinical relevance to the population at hand, based on a Neurosynth meta-analysis of different neuroimaging studies (13). Therefore, we carried out further SBC analyses with seed regions in the salience network and the motor cortex.

### Neurophysiological Differences between AMI and TA Subjects

Seed-to-whole-brain FC was estimated separately with each of the identified seeds, and between-group differences were inferred from cluster-level inferences (Table 1) obtained by thresholding voxel-based connectivity spatial parametric maps (p^unc^ < 0.001 cluster-defining threshold and p^FDR^ < 0.001 cluster-level threshold). With the salience network seed, three significant cluster-level inferences were made (Table. 1).

**Figure 3:**
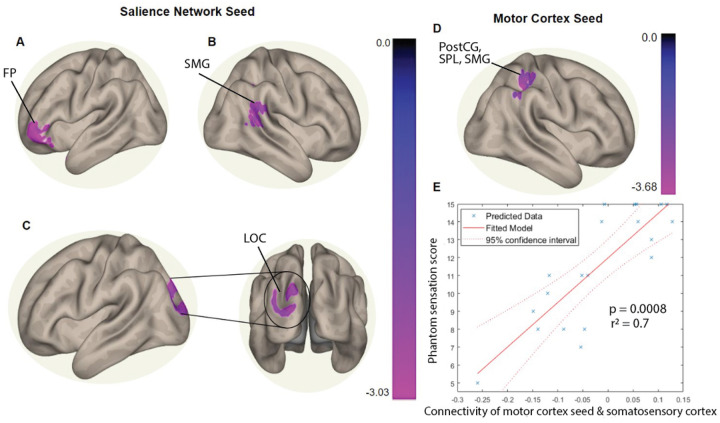
Results from the seed-based connectivity analysis with a between-subjects contrast of AMI > TA, controlling for age and head motion. The colorbar represents the Z-score detailing the extent of group differences in each cluster-level inference. Negative Z-scores indicate higher connectivity in the TA cohort. The **(A)** frontal pole cluster, **(B)** supramarginal gyrus cluster, and **(C)** occipital lobe cluster had stronger connectivity with the salience network seed in the TA group. **(D)** The dorsal attention cluster demonstrated stronger connectivity with the motor cortex seed in the TA group. **(E)** Multiple linear regression model (R^2^ = 0.7, p < 0.05) showing the significant association between phantom sensation scores and functional connectivity of the motor cortex seed with the somatosensory cortex in AMI and TA groups.

The largest cluster in the frontal pole (Fig. 3A) showed decreased FC with the salience network in AMI subjects compared to TA subjects. The second largest cluster (Fig. 3B) in the posterior supramarginal gyrus and the angular gyrus similarly showed decreased FC with the salience network in AMI (versus TA). Lastly, part of the occipital pole and lateral occipital cortex formed the third cluster, which exhibited significantly decreased FC with the salience network seed in AMI (versus TA) (Fig. 3C).

Furthermore, one significant cluster-level inference (p^unc^ < 0.001 cluster-defining threshold, p^FDR^ < 0.001 cluster-level threshold) was made when examining FC with the motor cortex seed in comparing AMI and TA groups. This cluster (Fig. 3D) was located on portions of the postcentral gyrus, superior parietal lobe and supramarginal gyrus, in a region that comprises the dorsal attention stream. Thus, the analysis illustrates decreased FC between the motor cortex seed and the dorsal attention stream in AMI subjects.

In addition to SBC analysis to find cluster-level differences between amputee groups, we explored whether we could fit a model to relate spontaneous FC to clinical correlates, non-painful phantom sensation scores, collected during the study. Specifically, a multiple linear regression was used to test if interregional FC in the primary sensorimotor cortex (between the primary motor cortex seed and somatosensory cortex) could significantly predict non-painful phantom sensation (Fig. 3E). The relationship between the motor cortex and somatosensory cortex was specifically chosen as a predictive component because according to the maladaptive plasticity model, aberrant wiring in the primary sensorimotor cortex as a result of the representation of the missing limb is thought to be a potential cause of phantom pain (14). Phantom sensations, both painful and non-painful, are largely associated with phantom pain, however the exact mechanism is unclear as there is variation in the experience of phantom sensation and its qualitative reports are difficult to quantify across studies (15, 16). Therefore, we chose to explore if using our rsfMRI data from both AMI and TA cohorts, the intrinsic connectivity of the sensorimotor cortex was associated with phantom sensation to the extent that a linear regression model could predict their relationship. The overall regression model was statistically significant (R^2^ = 0.7, F(20, 13) = 4.9, p < 0.05), suggesting that higher interregional connectivity in the sensorimotor cortex was coupled with higher phantom sensation.

### Reduction of Pathological Neural Signatures in AMI compared to TA Subjects

**Figure 4:**
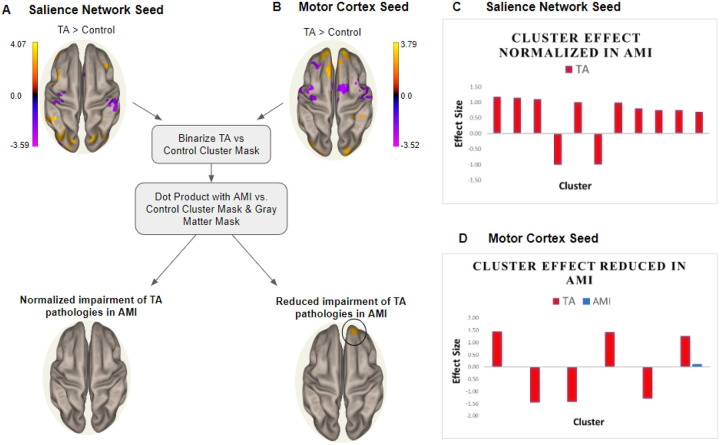
Testing whether there was a reduction of pathological neural signatures in AMI compared to TA. **(A)** The significant clusters (cluster-defining threshold: p^unc^ < 0.001, cluster-level threshold: pFDR < 0.001) from SBC analysis between TA and control groups. This SBC map was binarized and used to mask the SBC map containing cluster-level differences between AMI and control groups. The dot product was taken of the AMI-vs-control SBC map with the binary TA-vs-control mask and a binary gray matter mask. The result is shown in the SBC maps on the bottom row. **(B)** The same pipeline was repeated with the motor cortex seed. **(C, D)** The cohen’s *d* effect size of each cluster in TA-vs-control SBC map as well as with the AMI-vs-control SBC map after subsequent masking, represented in red and blue, respectively. **(C) N**o significant clusters were present with the salience network seed. **(D)** One cluster was present with considerably low effect size (shown in blue) with the motor cortex seed.

We hypothesized that there will be a significant reduction in neural impairments in AMI subjects compared to TA subjects. AMI subjects are less dependent than TA subjects on visual input during prosthetic control due to the preservation of proprioceptive afferents and also demonstrate less connectivity with salience network and dorsal attention stream regions, and so we presumed they would also have a corresponding reduction in pathological neural signatures. In other words, the goal of this experimental analysis was to determine whether the brains of AMI subjects maintained connectivities more similar to control subjects than TA subjects by examining the shift in neural signatures between the three groups. We designed an experiment (Fig. 4A, B) whereby we compared AMI and control groups, masked by the cluster-level difference map of TA vs. control comparison. That is, we tested if impairments seen in TA were being ‘normalized’ in AMI. There was either complete normalization or reduction of impairment in AMI subjects with both seeds, thus supporting our hypothesis. Specifically, with the salience network seed, no significant clusters were found in AMI compared to controls after masking with the TA-vs-control cluster mask (Fig. 4C). With the motor cortex seed, only one cluster remained in AMI compared to controls after masking with the TA-vs-control cluster mask. However, the effect size of this cluster drastically shrunk from what is often considered a large effect (d = 1.27) in TA-vs-control to a small effect (d = 0.21) in AMI-vs-control (Fig. 4D).

## Discussion

The goal of this study was to investigate group-level neurophysiological differences in FC between transtibial AMI and TA groups in order to obtain a mechanistic understanding of the brain network reorganization that occurs in the AMI paradigm. We hypothesized that the AMI surgery will induce reorganization of functional brain networks that both significantly varies from TA and also more closely resembles controls. We found evidence in support of our hypotheses. That is, we observed several key neurophysiological differences between AMI and TA subjects, as well as a reduction in presumably pathological neural signatures in those with AMI. Findings from this study provide researchers and clinicians with a critical mechanistic understanding of the effect of AMI amputation on brain networks even while not performing any extraneous task. Our findings provide neurobiological markers for assessing network reorganization after surgery, as well as identify important regions that have undergone rewiring post-operationally and their possible cognitive correlates that could be the focus of future neurorehabilitation efforts and research studies towards improved neuroprosthesis control.

To begin with, our results from the SBC analysis illustrated decreased connectivity between salience network and frontal pole regions in a map contrasting AMI and TA cohorts. Menon et al. (17) have demonstrated that the salience network is involved in the filtering and detection of salient stimuli, or in other words, deciding what to focus the brain’s limited perceptual and cognitive resources on based on the subset of sensory data available (18). The frontal pole where the first cluster was located, lies in the prefrontal cortex, which helps plan and organize movement, making decisions about which actions should be used for different situations (19). The lower connectivity between these regions may be related to evidence that AMI subjects rely less on visual streams during motor control, which could explain why areas functionally associated with choosing which salient stimuli to focus on and motor execution are less intrinsically coupled. We also observed a less coupling between the clusters in the visual cortex and the salience network in AMI subjects, which would corroborate prior evidence if AMI subjects do in fact assign less salience to the visual stream than TA subjects (20, 21, 22). Additionally, the cluster lying primarily on the supramarginal gyrus has decreased connectivity to the seed ROI within the salience network in AMI subjects. The supramarginal gyrus plays a role in interpreting sensory data and in the perception of space and limb location, as well as identifying postures and gestures of other people as part of the mirror neuron system (23, 24). Thus, the reduced coupling between these spatially distinct regions in AMI subjects may be motivation to explore possible neural correlates of fMRI connectivity to neural resource allocation while a subject is exposed to proprioceptive and sensory data, or while their mirror neuron system is activated in a comparison with TA subjects.

Furthermore, we observed decreased connectivity in AMI subjects between the motor seed ROI and a cluster that lies on several distinct regions that collectively comprise the dorsal attention stream. The dorsal attention stream is responsible for integrating information for immediate movements (25). As such, the relationship between these clusters may imply that AMI subjects consolidate less data while engaging in movements necessitating faster reaction times, which would make sense due to the restoration of afferent feedback and proprioception provided by this surgical paradigm. Research looking at cognitive load of amputees is important for prosthesis development and rehabilitation because the increased cognitive burden and neural fatigue associated with prosthesis use can lead to their abandonment (26). The neural fatigue brought on by excessive cognitive load is impacted by factors such as reduced proprioception and phantom sensations, maladaptive plasticity, and lack of embodiment, which are all induced by the changes in neural schema caused by amputation. In this research study, the reconfiguration of intrinsic brain networks, which prime task response, suggest that there may be a measurable reduction in cognitive burden during motor performance tasks in the AMI cohort compared to the TA cohort. To this effect, the current study provides motivation to more precisely study the impacts of the AMI surgery on cognitive burden and neural fatigue during prosthesis use and training.

Additionally, the neural signatures present in the SBC map contrasting TA and control groups were either normalized or significantly reduced after masking with the SBC map contrasting AMI and control groups. This finding suggests that subjects in the AMI cohort are alleviated of the neuropathologies that those in the TA cohort previously possessed, as the AMI cohort are overall more similar in neurophysiology to controls in terms of cluster-level differences. That is to say, AMI subjects still showed a reconfiguration in rsfMRI topology in relation to controls, yet to a significantly lesser extent than TA subjects. Some level of compensatory functional network reorganization is to be expected after something as physically traumatic as limb loss. Similar to TA subjects, AMI subjects showed differences in salience towards proprioception, as well as in integrating information from the mirror neuron system and sensory feedback for motor movement. Overall, the rsfMRI connectivity patterns in AMI subjects bear a closer resemblance to controls insofar as the coupling of dorsal attention and visual streams with regions functionalized for executing movement.

A strength of our approach is the use of the novel NORDIC denoising technique, which significantly reduced the thermal noise in functional images, increasing the statistical power of the data by a factor of 2–3. However, a limitation that needs to be recognized when assessing generalizability includes the sample size; however, at the time of data collection, the AMI amputation was only being performed by one surgeon in the world at Brigham and Women’s Hospital in Boston, MA, so the AMI cohort was as large as possible. Today, additional surgeons across the United States and the world are performing the AMI surgery, allowing future studies to rely on more surgically homogeneous cohorts.

To summarize, our findings suggest that the rsfMRI connectivity patterns following the AMI surgery may better lend themselves to improved prosthetic control and increased sense of artificial limb embodiment. That is, AMI subjects show significantly less rsfMRI coupling with regions functionally dedicated to selecting where to focus attention and what information to integrate when it comes to salient stimuli. In addition, AMI subjects show a reduction of neural signatures from a presumably pathological origin, and thereby cortical reorganization that bears a closer resemblance to the brains of control subjects than those of TA subjects. We also show FC between the primary motor cortex and the somatosensory BA3 region is a significant predictor of phantom sensation, which has been associated with prosthesis embodiment (27). These findings contribute a mechanistic understanding of FC differences between AMI and TA cohorts in the intrinsic brain networks that prime task response. These research contributions have implications for neurorehabilitation of persons with transtibial amputation and more broadly, as well as for clinical translation in the field of orthopedic surgery. For instance, this study could inform future research looking at the progression of plasticity for TA patients receiving an AMI revision surgery with regard to the effects on prosthetic device adaptation and utility. Furthermore, future research could examine the ways in which the AMI’s neurophysiological effects correlate with prosthesis control and represent embodiment, as well as how clinicians may be able to predict rehabilitation outcomes and adapt to improve them with prosthesis training (28, 29). Another interesting topic to investigate is the effect of non-invasive neurostimulation techniques on FC of the specified target regions that predict phantom sensation, and subsequently examine potential impacts on prosthesis control and embodiment in clinical studies (30). Future research on the neurophysiological impacts of the AMI surgical architecture should also extend these results across different combinations of agonist-antagonist muscle constructs in patients with both upper and lower limb amputation.

## Materials And Methods

### Participants and study design

The participants that underwent neuroimaging belonged to the following conditions: unilateral lower limb AMI amputees (n = 12), TA (n = 7), and biologically-intact controls (n = 10). Within the AMI cohort, the majority of the scans were on patients with a transtibial AMI amputation, save for one patient with a transfemoral AMI. Similarly, within the TA cohort, the majority of the scans were on patients with transtibial standard amputations, save for one patient with a transfemoral amputation. As the cortical representations for the thigh and calf regions of the lower leg are maintained within a 2–3 mm radius from each other, which is approximately one functional voxel, we determined the outlier was sufficiently negligible and categorized the cohort according to the majority, that is, transtibial amputees (31). To the best of our ability, TAs were selected to be amputation-to-scan-time-matched and age-matched to AMI amputees. The matching criteria prioritized amputation to scan time (± 13 month variance; SD, ± 6.15 months) and age (± 7 years variance; SD, 4.16 years), similar to that of prior studies that looked at neural plasticity following amputation (32, 33, 34). Further details about subject demographics, dominant side, amputation to scan time, age and gender can be found in Table S1. All participants were recruited in a nonblinded fashion and provided written informed consent prior to imaging. The protocol and procedures for functional neuroimaging were approved by the Mass General Brigham (formerly Partners HealthCare) Institutional Review Board (IRB; protocol no. 2017P002635).

The AMI amputation paradigm of the subjects in this study consisted of an AMI construct for both the subtalar and ankle joints (35, 36). The subtalar AMI construct mechanically linked the tibialis posterior and the peroneus longus muscle. The ankle AMI construct mechanically linked the lateral gastrocnemius to the tibialis anterior muscle. In each construct, a tendon harvested from the amputated ankle joint at the time of amputation was passed through a synovial canal and linked the muscle pair. The TA paradigm of the TA subjects in this study was the standard amputation that is performed in hospitals in the United States.

Phantom limb sensation was self-reported by individuals with AMI and TA while walking with a passive, conventional transtibial prosthesis, as well as while sitting with and without the prosthesis. In the self-report, patients were asked to describe the vividness of the perception of their heel pad, arch of the foot, balls of the footpad, and toes, after which these responses were marked according to a 1 to 3 scale. Then, these responses were normalized to produce a final phantom sensation score ranging from 0 to 15, with 15 being the highest sensation and 0 being no sensation.

### Data acquisition

Structural and functional MRI data were collected in a 3T scanner at the Athinoula A. Martinos Center for Biomedical Imaging at the Massachusetts General Hospital (MGH). The 3T Connectome scanner, based on the Siemens Skyra system, had the following customized parameters: Gmax = 300 mT/m “connectome” gradients and a slew rate of 200 mT/m/ms. Head motion was restricted by foam padding placed strategically inside the custom 64-channel array coil. Anatomical data was collected with a T1-weighted magnetization-prepared rapid acquisition gradient echo (MPRAGE) sequence with the following parameters: 1-mm isotropic resolution; 208 slices; flip angle, 7°; repetition time (TR), 2530 ms; echo time (TE), 1.61 ms; generalized autocalibrating partial parallel acquisition (GRAPPA) factor, 4; acquisition time, 3 min and 40 s. RsfMRI data were acquired using a 2D echo planar imaging (EPI) sequence with the following parameters: 2-mm isotropic voxels; 68 slices; flip angle, 41°; TR, 1080 ms; TE, 30 ms, 8-min run.

### Preprocessing and denoising

**Figure 5:**
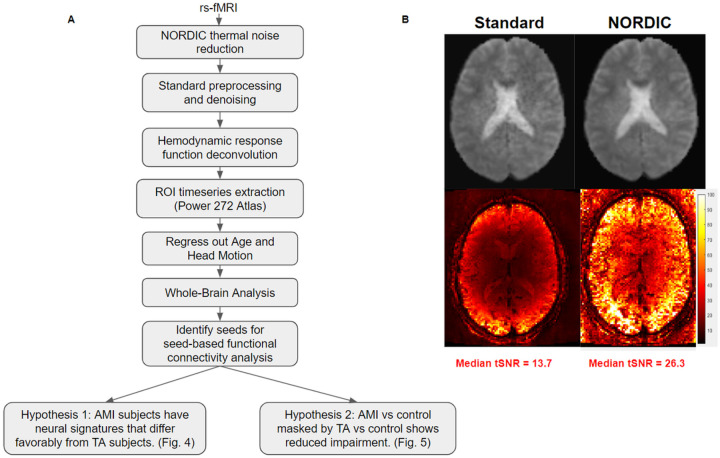
Analysis pipeline and results from NORDIC denoising. **(A)** Flowchart of the signal processing and analysis pipeline. **(B)** (Top) Example functional slice from subject 2 before and after NORDIC denoising. (Bottom) Temporal signal to noise ratio (tSNR) of the same slice from subject 2 before and after NORDIC denoising. Median tSNR after NORDIC increases by a factor of two compared to standard preprocessing before NORDIC, from 13.7 to 26.3.

An overview of the methodology applied to the raw rsfMRI data to arrive at the stage of answering our hypotheses based on group-level inferences is presented in Fig. 5A. At the onset, a novel signal processing approach called NOise Reduction with DIstribution Corrected (NORDIC) PCA was applied to the raw rsfMRI data (37). This denoising algorithm addresses the signal-to-noise and functional contrast-to-noise ratios by selectively suppressing the thermal noise contribution present in the fMRI data. In this case, the NORDIC technique reduced the noise by an average of 2–3 times per random voxel and doubled the median temporal signal to noise ratio (Fig. 5B), drastically increasing the statistical power of the data.

Afterwards, standard fMRI preprocessing steps were done in the CONN toolbox version 21, which is based on SPM12 (38): functional realignment, slice-timing motion correction, ART-based outlier identification, normalization to MNI152 standard space, and spatial smoothing (Gaussian filter with a 8mm FWHM). The residual BOLD signals were filtered through a 0.01 Hz high-pass filter in order to eliminate low-frequency drift often attributed to physiological noise or subject motion. Following this, linear detrending and denoising was done with 5 principle components and their derivatives of white matter (WM) and cerebrospinal fluid (CSF) signals, as well as 6 motion parameters and their derivatives. Quality control was done on the processed images and no subjects were removed. Quality control results such as those of motion correction and denoising are shown in Fig. S1. Any further denoising and analysis was performed in MATLAB (39). Estimation and deconvolution of the hemodynamic response function (HRF) was performed (40) to recover the rsfMRI signal without potential confounds from HRF variability on FC estimation (41) and FC group differences (42, 43).

### Structural image parcellation

Regional time series from functionally homogeneous ROIs were extracted from the deconvolved BOLD signals using the Power 272 Atlas, which remains a competitive parcellation performer using the distance-controlled boundary coefficient (44). The 272 ROIs are comprised of 242 cortical ROIs from the Power-Petersen atlas (45), 16 subcortical ROIs from the Harvard-Oxford structural atlas (46, 47, 48, 49) and 14 cerebellar ROIs from the Buckner cerebellar atlas (50).

### Exploratory whole-brain analysis

Since this was an exploratory analysis, a data-driven approach was used to identify which ROIs to use for SBC analysis. First, the FC, or Pearson’s zero-lag cross correlation coefficient, between each of the 272 ROIs was computed for each subject in each group. In the following statistical analyses, both head motion and age were extracted and utilized as covariates. Framewise displacement was calculated for each subject and subsequently used as the regressor representing head motion. Then, a three-way ANOVA test (p^unc^ < 0.001) was applied to find any significant differences between the AMI, TA or control groups. Finally, paired t-tests (p < 0.05, Bonferroni correction) were performed only within the significant correlations from the prior ANOVA test in order to find relevant pairwise correlations (Fig. 2A, B).

Among the results, an ROI in the salience network stood out as being associated with the highest node degree, as well as for being uniquely altered in the TA subjects. In other words, an ROI on the right mid-cingulum was involved in significant pairwise connections between both AMI and TA, as well as TA and control comparisons. This was not the case for any other ROIs. Thus, this salience network ROI (MNI coordinates: 5, 23, 37, spherical radius = 10 mm) was selected as a seed in further analysis. Additionally, an ROI in the medial primary motor cortex was chosen due to the prominent effects of functional reorganization following lower limb amputation, and their resulting clinical impact (51). The ROI was selected based on a meta-analysis identifying 438 neuroimaging studies that used the particular keywords “motor cortex” in NeuroSynth, a large-scale database of mappings between neural and cognitive states. This motor cortex seed (MNI coordinates: 4, −2, 54; spherical radius = 10 mm) is anatomically located superior and posterior to the salience seed. The BrainNet Viewer software was used for the visualization of the connectivity results from the exploratory whole-brain analyses, as well as for visualizing the selected seed ROIs in a brain volume (52).

### Seed-based connectivity analysis

The SBC analysis was performed using the CONN toolbox. To assess group level FC differences, a between-subjects contrast of [−1 1 0] was applied to the general linear model for each between-groups comparison, while simultaneously regressing out the effects of age. The SBC analysis was performed using both the ROI in the salience network and that in the primary motor cortex. The level of FC reported corresponds to the z-score, or the Fisher-transformed bivariate correlation coefficient, between the average of the BOLD timeseries within the ROI and the BOLD timeseries of each independent voxel. The statistically significant cluster-level inferences obtained were labeled according to the Harvard-Oxford and AAL atlases that are default in the CONN toolbox.

### Masking

An image masking technique was utilized to test the second hypothesis. To do this, a mask of the TA vs control clusters was extracted and binarized. This mask was coregistered (estimated and resliced) to fit into MNI space, and then its dot product was taken with a gray matter mask to eliminate any potential noise artifacts. In tandem, a mask of the AMI vs control clusters was extracted and coregistered, after which the dot product between it and the aforementioned “TA-control” mask was taken. Following this procedure, the effect size of the clusters before and after masking were analyzed and calculated in terms of Cohen’s d effect size for ease of interpretation (d = 0.2 is a small effect; d = 0.8 is a large effect).

### Multiple linear regression model predicting phantom sensation

A multiple linear regression analysis was used to test if FC between the motor cortex and the somatosensory cortex significantly predicted phantom sensation. The studies showing significant associations between phantom pain and phantom sensation were performed in a sample with upper limb amputee patients, however, we presume that the neural manifestations translate to lower limb amputee patients as well. The same logic applies for studies that developed the maladaptive plasticity theory. Furthermore, the somatosensory cortex consists of four Brodmann regions (1, 2, 3a, 3b), but in this case, only Brodmann area’s 3a and 3b were selected to represent the somatosensory cortex because only they were defined in the Power 272 atlas. Multiple (n = 6) ROIs comprise the Brodmann area 3 regions in the Power 272 atlas, all of which were used as predictive components in the multiple linear regression analysis.

## Figures and Tables

**Table 1.** Table of all the significant cluster-level differences (cluster-level threshold: p^FDR^ < 0.001, cluster-defining threshold: p^unc^ < 0.001) from the resting state SBC analysis. The top and bottom halves of the table contain ROIs in relation to their FC with the salience network and motor cortex seeds, respectively. Enumerated in the table is the region in which a given cluster is situated, based on the Harvard-Oxford atlas default in the Conn toolbox, each cluster’s centroid in MNI space, each cluster’s size, the cluster-level and cluster-defining p-value thresholds, as well as the Cohen’s *d* effect size.

Salience network seed						
ROI spherical radius = 10mm						
	Cluster Region	Centroid (MNI Space)	Cluster Size	Cluster-defining p-unc	Cluster-level p-FDR	Cohen’s *d* Effect Size
**AMI >TA**						
	FP, FO	MB, +32, −14)	369	0.000001	0	−1.09
	SMG, AG	(+46, −38, +28)	227	0.000259	0.000024	−1.17
	OP, LOC	(−26, −86, +32)	218	0.000694	0.000026	−0.95
**AMI > Control**						
	LOC, TOFusC, OFusG, Cereb	(+46, −70, −06)	360	0.000017	0	−0.97
	SMG, PostCG, PO	(−56, −22, +30)	337	0.0002	0	−1
	LOC	(−50, −80, +02)	177	0.00022	0.00023	−0.97
	PP, IC, CO	(−50, +00, −04)	149	0.00038	0.00093	−1.12
	SMG. PO	(+60, −26, +40)	145	0.00035	0.0009S	−0.88
**TA > Control**						
	Forb, TP, FP	(−48, +32, −14)	306	0.00001	0.000001	1.19
	LOC, OP	(−20, −86, +38)	235	0.00049	0.000015	1.15
	MidFG, PreCG	(−44, +10, +44)	162	0.00015	0.00057	1.1
Motor cortex seed						
ROI spherical radius = 10mm						
**AMI > TA**						
	PostCG, SPL, SMG	(+38, −32, +54)	211	0.00005	0.000114	−1.78
**AMI > Control**						
	PreCG, PostCG, TP	(−56, +02, −06)	370	0.00002	0	−1.1
	PreCG, PostCG	(+56, −02, +46)	279	0.000013	0.000002	−1.19
	PreCG	(−10, −16, +68)	194	0000007	0.000089	1.25
	SMG, PostCG	(+60, −22, +38)	174	0.0003	0.00021	−1.2
**TA > Control**						
	FP, PaCiG, SFG	(−18, +58, +24)	340	0.000017	0	−0.71
	OP, LOC	(+24, −94, +20)	199	0.000011	0.00011	−1.46
	SFG	(−04, +36, +50)	184	0.000023	0.00017	1.42
	FP	(+18, +62, +24)	174	0.000003	0.00023	1.27
	LOC	(−56, −68, +08)	160	0.00037	0.00042	1.01
	AC, SMA, PaCiG	(+10, +08, +46)	146	0.000003	0.00081	−1.05

Abbreviations: FP = frontal pole, FO = frontal operculum, SMG = supramarginal gyrus, AG = angular gyrus, OP = occipital pole, LOC = lateral occipital cortex, TOFusC = temporal occipital fusiform cortex, OFusC = occipital fusiform gyrus, Cereb = cerebellum, PostCG = postcentral gyrus, PO = parietal operculum, PP = planum polare, IC = insular cortex, CO = central operculum, Forb = frontal orbital cortex, TP = temporal pole, MidFG = middle frontal gyrus, PreCG = precentral gyrus, SPL = superior parietal lobe, PaCiG = paracingulate gyrus, SFG = superior frontal gyrus, AC = anterior cingulate.

## Data Availability

Study data may be obtained from the corresponding author upon reasonable request.
